# Photobiomodulation regulates adult neurogenesis in the hippocampus in a status epilepticus animal model

**DOI:** 10.1038/s41598-022-19607-5

**Published:** 2022-09-09

**Authors:** Namgue Hong, Gi Won Kang, Ji On Park, Phil-Sang Chung, Min Young Lee, Jin-Chul Ahn

**Affiliations:** 1 Medical Laser Research Center, Dankook University, Cheonan, Republic of Korea; 2 Department of Biomedical Science, College of Medicine, Dankook University, Cheonan, Republic of Korea; 3 Department of Medical Laser, Graduate School, Dankook University, Cheonan, Republic of Korea; 4 Beckman Laser Institute Korea, Dankook University, Cheonan, Republic of Korea; 5 Department of Otolaryngology-Head and Neck Surgery, College of Medicine, Dankook University, Cheonan, Republic of Korea

**Keywords:** Optics and photonics, Lasers, LEDs and light sources, Other photonics, Diseases of the nervous system

## Abstract

Status epilepticus (SE) refers to a single seizure that lasts longer than typical seizures or a series of consecutive seizures. The hippocampus, which is vulnerable to the effects of SE, has a critical role in memory storage and retrieval. The trisynaptic loop in the hippocampus connects the substructures thereof, namely the dentate gyrus (DG), CA3, and CA1. In an animal model of SE, abnormal neurogenesis in the DG and aberrant neural network formation result in sequential neural degeneration in CA3 and CA1. Photobiomodulation (PBM) therapy, previously known as low-level laser (light) therapy (LLLT), is a novel therapy for the treatment of various neurological disorders including SE. However, the effects of this novel therapeutic approach on the recovery process are poorly understood. In the present study, we found that PBM transformed SE-induced abnormal neurogenesis to normal neurogenesis. We demonstrated that PBM plays a key role in normal hippocampal neurogenesis by enhancing the migration of maturing granular cells (early neuronal cells) to the GCL, and that normal neurogenesis induced by PBM prevents SE-induced hippocampal neuronal loss in CA1. Thus, PBM is a novel approach to prevent seizure-induced neuronal degeneration, for which light devices may be developed in the future.

## Introduction

Status epilepticus (SE) refers to a single seizure that lasts longer than typical seizures or a series of consecutive seizures. The non-return to baseline status seen in SE causes rapid and widespread neuronal damage. The degree of damage depends on seizure severity and duration. Dysfunctional electrical activity of the central nervous system is associated with neurodegeneration, abnormal neurogenesis in the hippocampus, and behavioral and cognitive deficits^1,2^.

The hippocampus, which is vulnerable to the effects of SE, has a critical role in memory storage and retrieval. The trisynaptic loop in the hippocampus connects the substructures thereof, namely the dentate gyrus (DG), CA3, and CA1. The entorhinal cortex provides input to the DG (synapse 1), which in turn provides input to the CA3 through the mossy fiber pathway (synapse 2). CA3 provides input to the CA1 through the Schaffer collateral pathway (synapse 3). Finally, the CA1 is connected to the entorhinal cortex, thereby completing the trisynaptic loop^3^. The trisynaptic loop is implicated in the pathomechanism of hippocampal damage during SE.

In an animal model of SE, excitotoxic changes in hippocampal substructures were demonstrated. Pilocarpine-induced acute seizures strongly induce abnormal hippocampal neurogenesis. These abnormal changes are characterized by increased proliferation of neural progenitor cells and abnormal integration of the newly generated granular cells in the subgranular zone (SGZ) of the DG^4,5^. Aberrant integration caused by the newly generated neurons is termed mossy fiber sprouting. The mossy fibers extend into the hilus and project to excitatory (mossy cells) and inhibitory interneurons. Then, the fibers pass through the stratum lucidum and synapses of CA3 pyramidal neurons^6^. In the pilocarpine model, mossy fiber sprouting due to spontaneous seizures disrupts the synapses at CA3 and induces loss of cells in the CA3 and CA1 pyramidal cell layers. In brief, abnormal neurogenesis in the DG and aberrant neural network formation results in sequential neural degeneration in CA3 and CA1.

Several new antiepileptic drugs have been introduced in recent years^7^; however, most of them only have anticonvulsant effects; neuroprotective effects are very limited. Antiepileptic drugs fail to control seizures in 20–30% of patients^8,9^. Therefore, new alternatives to control epilepsy development are required. Photobiomodulation (PBM) therapy, previously known as low-level laser (light) therapy (LLLT)^10^, is a novel therapy for the treatment of various neurological disorders. Emerging evidence suggests that PBM protects neurons in epilepsy models, including an SE model^11–14^. In addition, PBM therapy causes hippocampal alterations^15^. However, the effects of this novel therapeutic approach on the recovery process are poorly understood. Based on previous studies that reported increased stem cell and progenitor cell proliferation with PBM therapy^16^, its effects on DG neurogenesis, and on CA3 and CA1, should be elucidated. Furthermore, recent studies have suggested that the functional connectivity among hippocampal substructures is highly complex, and not limited to a one-way loop (e.g., there is a direct connection between the entorhinal cortex and CA3 or CA1). Therefore, PBM may not reverse the pathomechanism underlying the excitotoxicity caused by SE.

In the present study, we administered PBM therapy to the depilated head of an SE animal model, allowing a portion of the light energy to reach the hippocampus. The population of mature neurons in DG was compared between the PBM therapy and SE only groups. In addition, the population of premature cells and their morphological changes were compared to evaluate the process of neurogenesis in detail. Finally, we evaluated the effect of PBM on sequential neuronal degeneration in CA3 and CA1.

## Results

### Hilar interneuron cell population in the DG in an SE animal model with or without PBM

**Figure 1:**
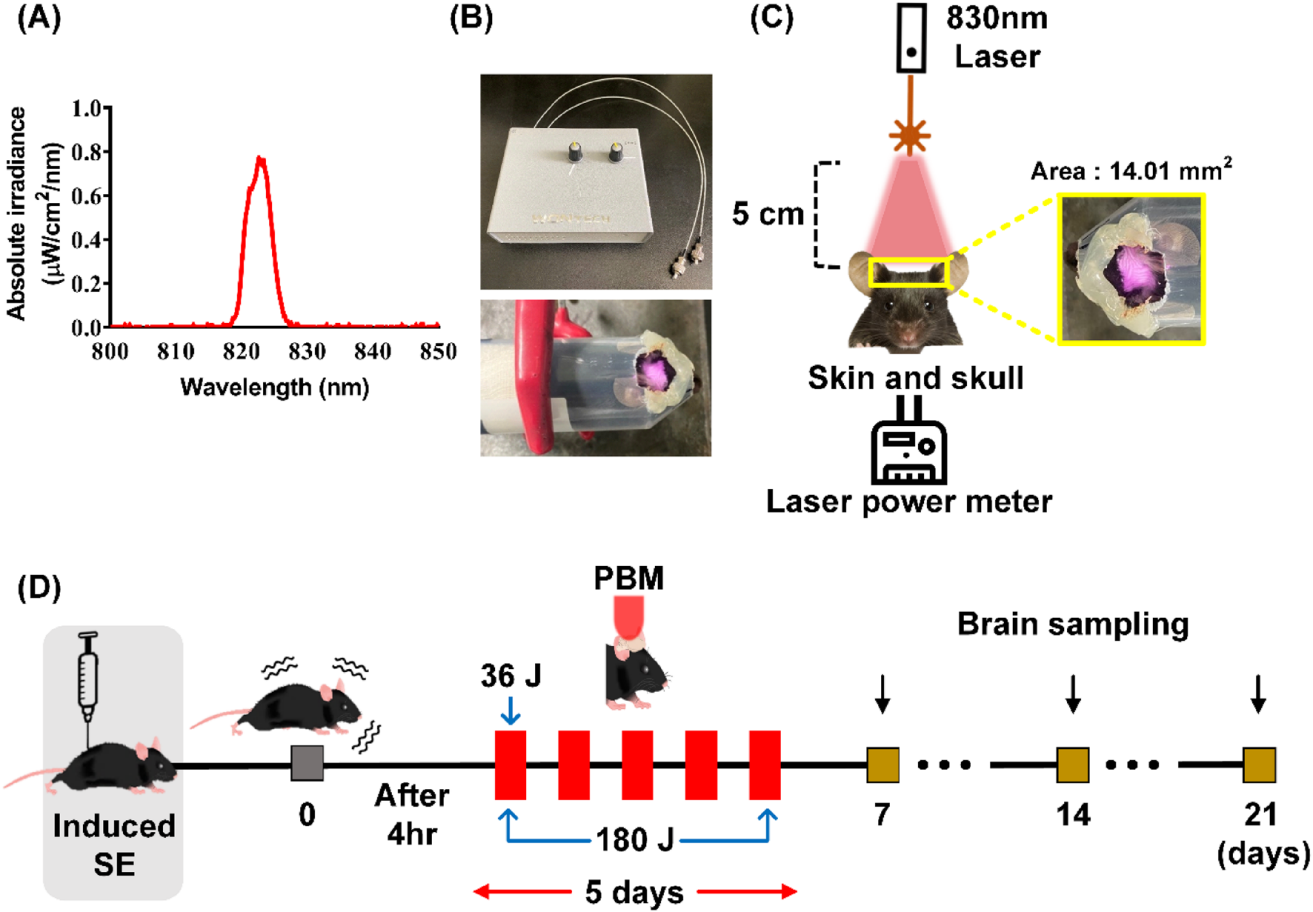
PBM irradiation and experimental schedule. (**A**) Beam profiling of PBM using a spectrophotometer. An 830 nm laser with specific narrow wavelength was used to irradiate the SE mouse model. (**B**) Experimental setup for treatment with PBM. After the hair on the head was depilated, the mouse was placed in the 50 ml tube with a hole in the head position to fix the movement, and then an 830 nm laser was used for irradiation for 12 min at a distance of 5 cm with a power of 50 mW/cm^2^. (**C**) The scalp was irradiated with an area of 14.01 mm^2^, and laser transmission were measured by power-meter detector. (**D**) 830 nm laser was irradiated at 4 h after SE-induction only once as 36 J or during 5 days as total 180 J. Then, brains were collected for the epifluorescence analyses on days 7, 14, or 21. This figure was generated with Microsoft Visio version 2016 (Microsoft, Redmond, WA, USA).

**Figure 2:**
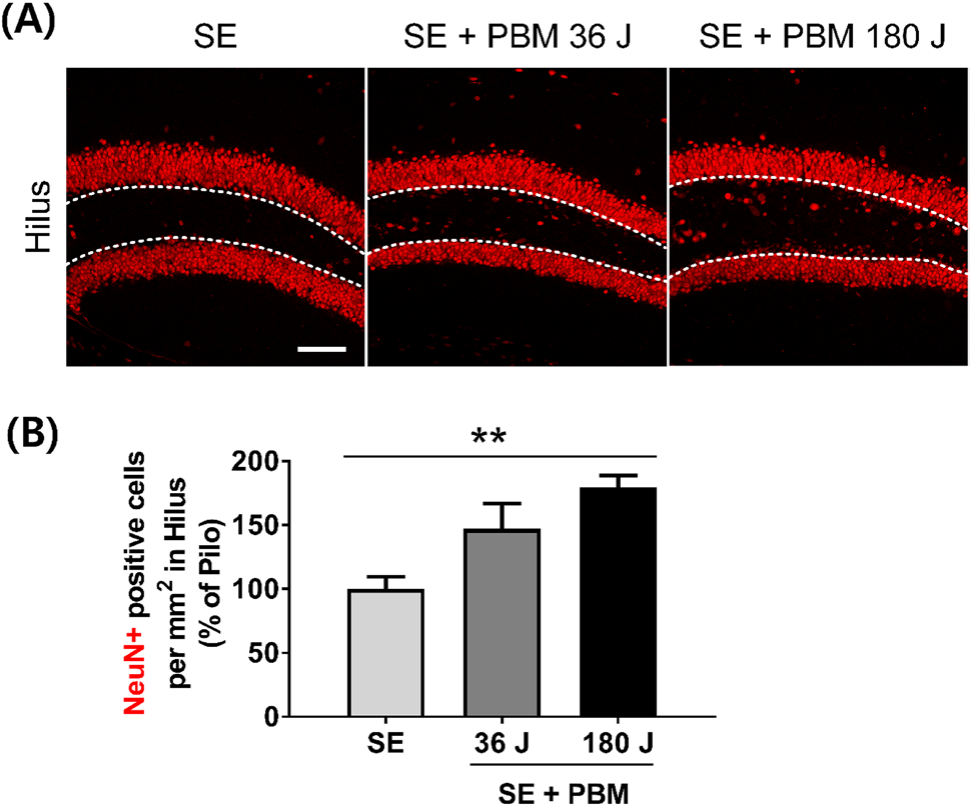
PBM upregulated neuronal population in hilus after SE. (A) Representative coronal section views of Z-stacks obtained by confocal microscopy show the localization of NeuN (for mature neuron, red) positive cells in hilus (area between dotted line) on the dentate gyrus of the hippocampus. Scale bar 100 µm. (**B**) Quantification of the number of mature neurons (NeuN) in hilus are shown. The treatment of 180 J PBM shows statistically larger population of hilar interneurons compared to SE only at day 21 after induced SE. The quantitative data are presented as the mean ± SEM. **P < 0.01. This figure was generated with Microsoft Visio version 2016 (Microsoft, Redmond, WA, USA).

Mice were injected with pilocarpine (320 mg/kg) to induce SE with a minimal mortality rate. The loss and neurogenesis of hilar interneurons are key events in the seizure-like discharges that cause further neuronal damage^17^. We evaluated the population of neuronal cells in this anatomical area 21 days after pilocarpine injection. Patients were treated with PBM for 1 (single, 36 J) or 5 (multiple, 180 J) consecutive days following 4 h of pilocarpine injection (Fig. 1D). Compared to the SE only group, the SE + 180 J PBM group showed higher population of NeuN, a mature neuronal marker (Fig. 2). However, there was no difference in the NeuN population between the SE only and SE + 36 J PBM groups (data not shown). The difference between SE only and SE + 180 J PBM groups was 179.8 ± 8.9% in the ANOVA test (F = 8.45, p = 0.0017) followed by Bonferroni’s post hoc test. The results suggest that PBM 180 J exposure increased the hilar interneuron population in the DG after pilocarpine-induced seizures.

### Proliferating cell/neuron population at the hilus in the DG of an SE animal model with or without PBM

**Figure 3:**
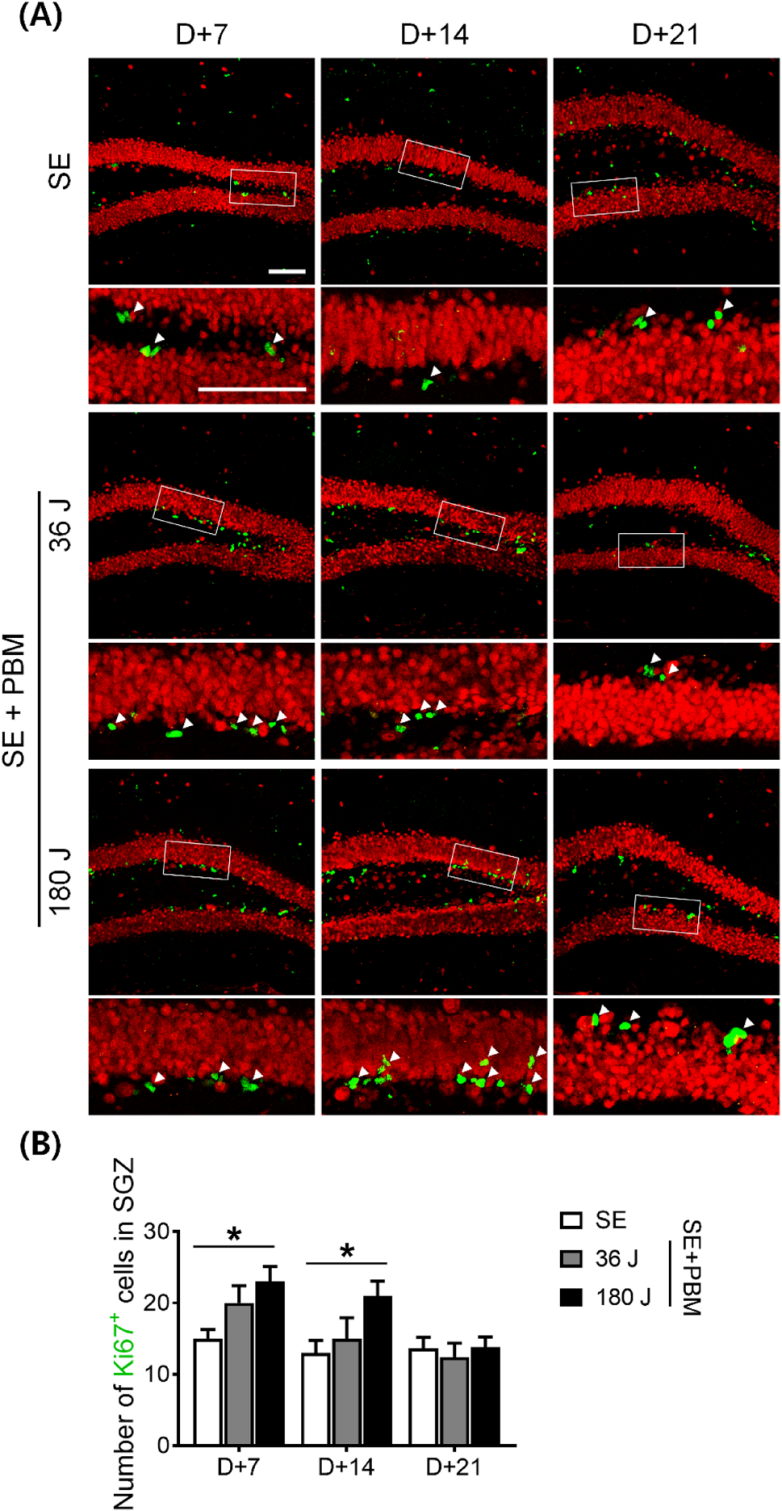
PBM increased cell proliferation in the SGZ of the DG on the hippocampus after SE. (**A**) Representative coronal section views of Z-stacks obtained by confocal microscopy show the localization of Ki67 positive cells (white arrow head, proliferated cells) in the SGZ of the DG. Scale bar 100 µm. Immunostaining was performed with NeuN (for mature neuron, red) and Ki67 (for proliferating cell, green). The insets are enlarged images of the boxed region. Scale bar 100 µm. (**B**) Quantification of the number of proliferated cells in the SGZ is shown. In the SGZ, proliferated cells increased in the PBM treated groups compared to the SE group in 180 J PBM at 7 and 14 days not at 21 day. The quantitative data are presented as the mean ± SEM. *P < 0.05 compared with to SE group. This figure was generated with Microsoft Visio version 2016 (Microsoft, Redmond, WA, USA).

**Figure 4:**
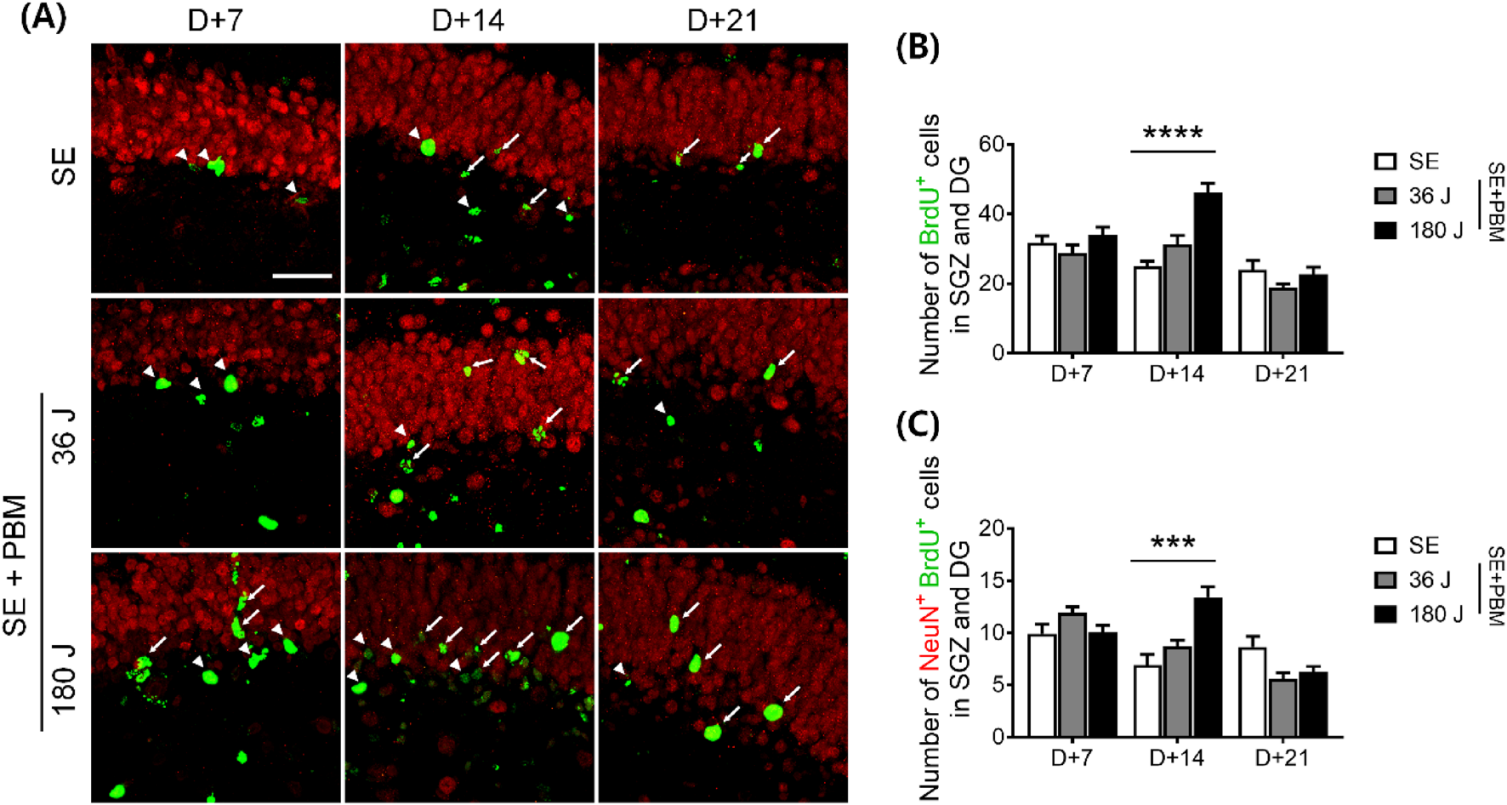
PBM increased newly generated/proliferating neurons in the SGZ and DG of the hippocampus after excitotoxicity by SE. (**A**) Representative coronal section views of Z-stacks obtained by confocal microscopy show the localization of BrdU and NeuN positive cells (white arrow head, BrdU-positive only; white arrow, BrdU and NeuN double-positive cells) in the SGZ and DG of the hippocampus. Scale bar 50 µm. Immunostaining was performed with NeuN (for mature neuron, red) and BrdU (for newly generated cell, green). (**B**) Quantification of the number of newly generated cells in the SGZ and DG is shown. Only BrdU-positive cells increased in the SGZ and DG of the hippocampus in the PBM treated groups compared to the SE group in 180 J PBM at 14 days. The quantitative data are presented as the mean ± SEM. ****P < 0.0001 compared with to SE group. (**C**) Quantification of the number of newly generated/proliferating neurons in the SGZ and DG is shown. NeuN and BrdU double positive cells increased of the hippocampal DG in the PBM treated groups compared to the SE group in 180 J PBM at 14 days. The quantitative data are presented as the mean ± SEM. ***P < 0.001 compared with to SE group. This figure was generated with Microsoft Visio version 2016 (Microsoft, Redmond, WA, USA).

It is important to identify the newly generated/proliferating cells in the DG because these cells are produced through neurogenesis, which could be responsible for the cascade of events that trigger excitotoxicity or the regeneration process required for recovery. Ki-67 is a marker of cell division, and Ki-67-positive cells were counted at three time points in this study (i.e., 7, 14, and 21 days after SE) (Fig. 3A). At 7 and 14 days, Ki-67-positive cell expression levels were higher in the 180 J PBM group (7 days: 23.3 ± 2.1; 14 days: 20.6 ± 2.1) compared to the SE only group (7 days: 14.7 ± 1.3; 14 days: 13.1 ± 1.8) according to ANOVA (7 days: F = 4.989, p = 0.0126; 14 days: F = 3.785, p = 0.0299) followed by Bonferroni’s post hoc test (Fig. 3B). At 21 days, no difference was observed in Ki-67-positive cell expression between the groups. We administrated BrdU to mice for 3 consecutive days before inducing SE, and evaluated the number of BrdU-positive cells in the DG (Fig. 4A). BrdU-positive cells were counted at three time points (i.e., 7, 14, and 21 days after SE). At 7 days, there was no difference in BrdU-positive cells between the groups. At 14 days, 180 J PBM showed higher numbers of BrdU-positive cells (46.0 ± 2.8) compared to the SE only group (25.0 ± 1.4) according to ANOVA (F = 27.05, p < 0.0001) followed by Bonferroni’s post hoc test (Fig. 4B). At 21 days, there was no difference in BrdU-positive cell expression between the groups. Then, we measured the co-expression of NeuN and BrdU, which reflects newly generated neurons. At 14 days, the number of NeuN-positive cells co-labeled with BrdU was higher in the 180 J PBM group (13.4 ± 1.0) compared to the SE alone group (6.9 ± 1.0) according to ANOVA (F = 10.41, p = 0.0003) followed by Bonferroni’s post hoc test (Fig. 4C). However, at 7 and 21 days, no difference was observed between the groups. Taken together, these results suggest that at all three time points after SE, newly generated/proliferating neurons (NeuN + /BrdU +) and non-neuronal cells (e.g., undifferentiated cells and glial cells) were observed in the DG. At higher doses of PBM, the number of proliferating cells was increased at 7 and 14 days. At 14 days, neuronal proliferation (NeuN + /BrdU +) was increased; however, at 7 days, total cell proliferation (Ki-67 + cells) was increased without an increase in neuronal proliferation (NeuN + /BrdU +). Therefore, neurogenesis likely occurs between 7 and 14 days after PBM. Detailed analysis of the undifferentiated and progenitor cell (early neuronal) populations and morphologies at these time points is necessary.

### Undifferentiated/early neuronal cell population in the hilus in the DG of an SE animal model with or without PBM

**Figure 5:**
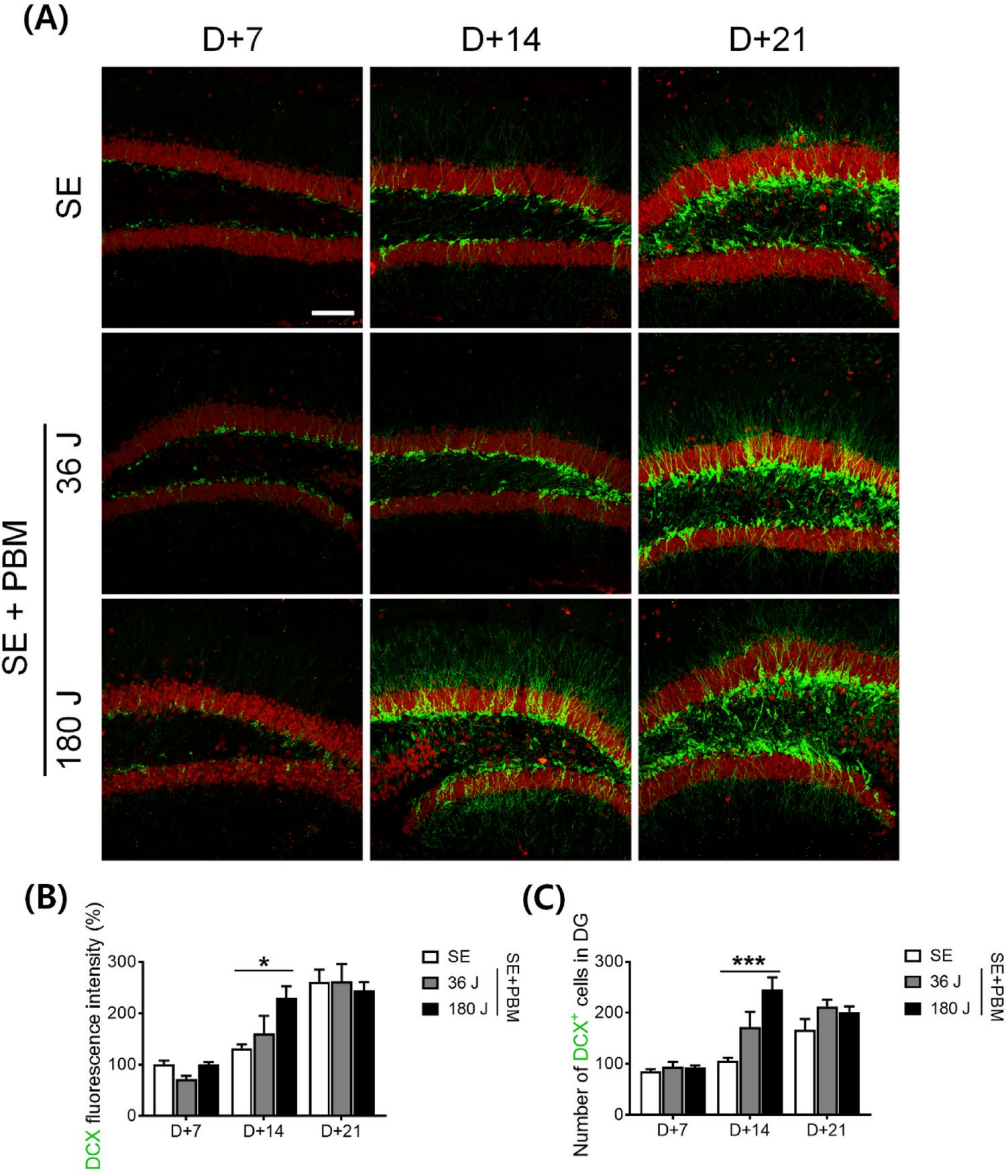
PBM increased undifferentiated/early neuronal cell population in the DG of the hippocampus after SE. (**A**) Representative coronal section views of Z-stacks obtained by confocal microscopy show the localization of DCX positive cells in the SGZ of the hippocampal DG. Immunostaining was performed with NeuN (for mature neuron, red) and DCX (for immature neuron, green). Scale bar 100 µm. (**B**) Quantification of the DCX fluorescent intensity in SGZ are shown. Expression of DCX fluorescent intensity are increased in 180 J PBM treated groups compared to the SE group only at 14 day. The quantitative data are presented as the mean ± SEM. *P < 0.05 compared with to SE group. (**C**) Quantification of the DCX positive cells in SGZ are shown. DCX positive cells are increased in 180 J PBM treated groups compared to the SE group only at 14 day. The quantitative data are presented as the mean ± SEM. ***P < 0.001 compared with to SE group. This figure was generated with Microsoft Visio version 2016 (Microsoft, Redmond, WA, USA).

To determine whether PBM influences the growth and maturation of new cells in the DG, we performed immunohistochemical staining using anti-DCX, a microtubule-associated phosphoprotein expressed by the vast majority of BrdU + cells, and by cells co-expressing early neuronal antigens, but not by antigens specific to glia or undifferentiated cells^18^. At 7 days after PBM exposure, the intensity and number of DCX + cells in the DG did not change compared to SE alone (Fig. 5A). Similarly, at 21 days after PBM treatment, the number of DCX + cells and staining intensity did not differ compared to SE alone. However, at 14 days after 180 J PBM treatment, the DCX staining intensity was increased by 229.9 ± 22.5% compared to SE alone (Fig. 5B), as confirmed by ANOVA (F = 5.213, p = 0.0104) followed by Bonferroni’s post hoc test. The number of DCX + cells was also significantly increased by 244.9 ± 24.1% after 180 J PBM exposure compared to SE alone, according to ANOVA (F = 10.41, p = 0.0003) (Fig. 5C) followed by Bonferroni’s post hoc test. These results suggest that the proliferation of early and mature neuronal cells occurs at the same time point (i.e., 14 days after SE), as evidenced by the proliferation of both early and mature neuronal cells. In addition, these results indicate that the proliferation of non-neuronal cells at 7 days may be due to the proliferation of precursor cells or glial cells.

### Early neuronal cell morphology (migration) in the hilus in the DG of an SE animal model with or without PBM

**Figure 6:**
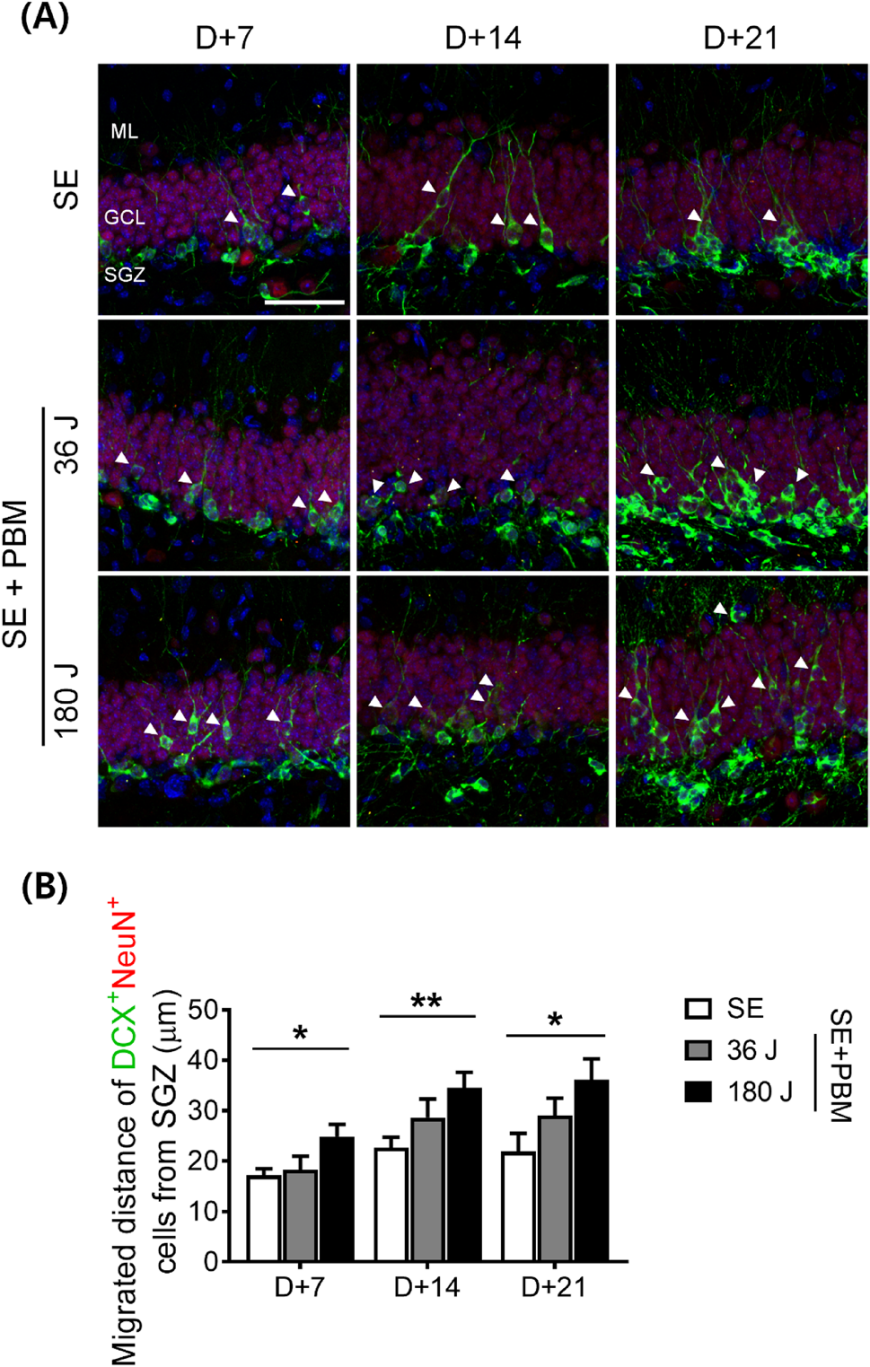
PBM increased early neuronal cell migration from the SGZ to the GCL of the hippocampus after SE. (**A**) Representative coronal section views of Z-stacks obtained by confocal microscopy show the localization of DCX positive cells in the SGZ of the hippocampal dentate gyrus. Immunostaining was performed with DAPI (for nucleus, Blue), NeuN (for mature neuron, red), and DCX (for immature neuron, green). The white arrowhead indicates the appropriate migrating DCX positive cells from the SGZ to the GCL. Scale bar, 100 µm. (**B**) Quantification of the migration distance of DCX positive cells are shown. 180 J PBM treatment statistically increased migration distance compared to SE only at all time points. The quantitative data are presented as the mean ± SEM. *P < 0.05 and **P < 0.05 compared with to SE group. This figure was generated with Microsoft Visio version 2016 (Microsoft, Redmond, WA, USA).

During hippocampal neurogenesis, maturing granular cells migrate from the SGZ to the granular cell layer (GCL), which is crucial because improper neuronal location due to incomplete migration may lead to aberrant neuronal sprouting and, consequently, excitotoxicity^4^. To determine the effect of migration on the GCL in response to PBM during maturation, we evaluated the distance of DCX + cells from the GCL. Overall, the migrated distance gradually increased from 7 to 14 and 21 days in the PBM 180 J-treated groups (Fig. 6A,B). At 7 days, the migrated distance was significantly increased in the SE + 180 J PBM group (24.8 ± 2.4 µm) compared to the SE group (17.2 ± 1.3 µm), according to ANOVA (F = 4.189, p = 0.0231) followed by Bonferroni’s post hoc test. In particular, at 14 days, the migrated distance was longer in the SE + 180 J group (34.6 ± 3.0 µm) compared to the SE alone group (22.6 ± 2.1 µm), according to ANOVA (F = 5.001, p = 0.0121) followed by Bonferroni’s post hoc test. We also calculated the migrated distance of DCX + cells at 21 days after PBM 180 J treatment, and found a significant increase (of 36.1 ± 4.2 µm) in the SE + 180 J PBM group compared to the SE group (21.9 ± 3.6 µm) according to ANOVA (F = 3.474, p = 0.0481) followed by Bonferroni’s post hoc test. These results show that PBM enhances the migration of maturing granular cells (early neuronal cells) to the GCL. Enhanced migration was observed at all time points, while increased proliferation was limited to the 14-day time point. This robust migration process might be crucial for transforming abnormal neurogenesis into appropriate neurogenesis, and for restoring the neuronal anatomical structure in the hippocampus.

### Neuronal cell population in CA3 and CA1 in an SE animal model with or without PBM

**Figure 7:**
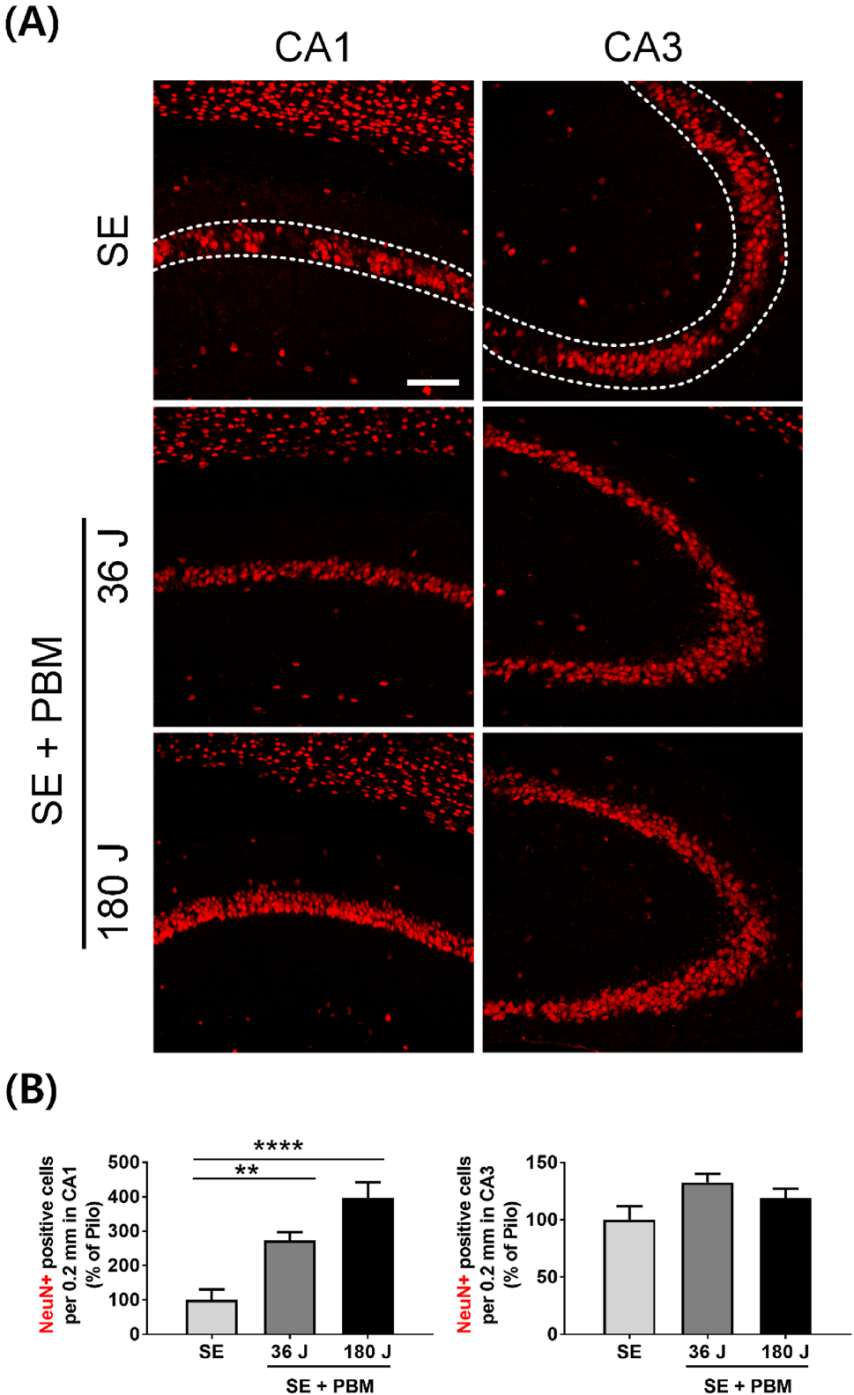
Appropriate neurogenesis by PBM triggers to survival of CA1 pyramidal neurons after SE. (**A**) Representative coronal section views of Z-stacks obtained by confocal microscopy show the localization of NeuN (for mature neuron, red) positive cells in CA1 and CA3 (area between dotted line) of the hippocampus. Scale bar 100 µm. (**B**) Quantification of the number of survival neurons (NeuN) in CA1 or CA3 are shown. The treatment of 180 J PBM shows statistically larger population of NeuN positive cells compared to SE only at day 21 after induced SE. The quantitative data are presented as the mean ± SEM. **P < 0.01, ****P < 0.0001. This figure was generated with Microsoft Visio version 2016 (Microsoft, Redmond, WA, USA).

After abnormal neurogenesis, which begins in the DG, degeneration of other substructures of the hippocampus, such as CA3 and CA1, occurs. In the present study, we observed a mature neuronal population in CA3 and CA1 after SE, with or without PBM, at 21 days. As expected, neuronal loss was observed in CA3 and CA1 in the SE animal model (Fig. 7). The cellular continuity of mature neurons was incomplete in CA3 and CA1. On the other hand, in both PBM groups, the cellular continuity of mature neurons was relatively well-established in both CA3 and CA1. Statistical analysis of NeuN-positive cells showed a significant difference among the groups (SE, PBM 36 J, and PBM 180 J), with higher numbers of cells seen in both PBM groups compared to the SE only group (PBM 36 J, 273.7 ± 23.6%; PBM 180 J, 397.1 ± 45.7%) in CA1 according to ANOVA (F = 16.84, p < 0.0001) followed by Bonferroni’s post hoc test. However, there was no significant difference between the groups (SE, PBM 36 J, and PBM 180 J) in the number of cells in CA3. These results indicate that abnormal neurogenesis and aberrant sprouting in DG after SE may lead to neuronal degeneration in both CA3 and CA1. Compared to the SE only group, the SE with PBM groups (especially in CA1) had relatively well-preserved neuronal populations, and greater neurogenesis in the DG, which may be explained by the robust migration of early neuronal cells to the GCL in the PBM groups.

## Discussion

Based on the evidence that PBM promotes cell proliferation and differentiation, and that abnormal neurogenesis occurs in the hippocampus after SE, we hypothesized that PBM might play a modulatory role in abnormal hippocampal neurogenesis after SE. We found that PBM transformed SE-induced abnormal neurogenesis to normal neurogenesis. Therefore, PBM is an important modulator of neurogenesis in the hippocampus after SE, which may aid understanding of post-SE hippocampal pathological changes and the development of a potential therapeutic approach for SE.

Hilar interneurons guide the maturation and integration of early neuronal cells, and are one of the primary cell types that degenerate during SE^19,20^. Initially, we measured the cell survival of hilar interneurons after SE and found that higher-energy irradiation of PBM increased NeuN-positive hilar interneuron cell survival at 21 days. This increase in the neuronal population could be due to the robust neurogenesis induced by PBM. Previous studies indicated that PBM can increase neurogenesis by stimulating the proliferation and differentiation of neuroprogenitor cells in a traumatic brain injury model^21,22^. In the current study, PBM significantly increased the number of BrdU/DCX/Ki67-positive cells in the DG at 14 days after SE, suggesting the augmentation effects of PBM on neurogenesis. Although further evidence is needed to confirm the role of the increase in neuroprogenitor cells induced by PBM, the presence of DCX- and NeuN-positive cells suggests that neuroprogenitor cells stimulated by PBM may play an important role in enhancing hippocampal adult neurogenesis.

The ectopic migration of early neuronal cells is a key feature of SE-induced neurogenesis^4^. In the DG of adult rodents, most seizure-related early neuronal cells showed ectopic migration and did not integrate into the hilus or migrate toward the hilar/CA3 border^23,24^. In the present study, we found increased migration of early neuronal cells to the GCL after PBM compared to SE alone at all time points (i.e., 7, 14, and 21). The increased migration of early neuronal cells to the appropriate location (i.e., GCL) by PBM suggests that aberrant sprouting can be minimized by preventing neuronal differentiation and/or axon sprouting at improper locations. This study demonstrated that the neurogenesis-modulating effect of PBM protected the loop synapses to CA1 and CA3 (particularly CA1), such that the neurons were preserved on day 21 after SE.

In the present study, the neuroprotective effect of PBM was significant in CA1, but not CA3. Although the reason for this difference is not clear, there are several possible reasons. One of them, there may be a direct pathway between DG and CA1, outside of the already known loop synaptic pathway. Therefore, PBM may have a robust influence on the previously known synapse pathway from DG to CA1 via the entorhinal cortex^3^.

Many studies have reported improvement in cognitive function in various neurodegenerative diseases after PBM^25–27^; therefore, PBM irradiation may also improve behavioral and cognitive function in the SE model. In the present study, the behavioral test (shown in our supplementary data S1) investigated whether PBM alleviated the symptoms of depression or anxiety induced by SE. There were result showing limited improvement only in high energy PBM. However, further studies with more detailed analysis of animal behavior regarding cognitive function is necessary.

PBM irradiation may cause scalp heating^28^; however, the thermal effects of PBM were not evaluated in this study. The photothermal effects of PBM are less likely to occur in brain tissue due to poor heat penetration into the brain^29^. In addition, we speculate that the heat at the skin and scalp did not modulate the therapeutic effect of PBM due to cytochrome c oxidase and cerebral hemodynamics^28^. However, the thermal effect should be evaluated in future studies, which should include histopathological examinations of the rat brain, particularly the hippocampus, as well as Nissl staining to monitor neuronal damage, TUNEL assay for assessing apoptosis, propidium iodide staining for analyzing necrosis, and electron microscopy to examine mitochondrial morphology. Finally, the synergistic anticonvulsive effects of adjunctive PBM with antiepileptic drugs should be evaluated in future studies. To allow the use of PBM therapy in epilepsy patients, studies in human cells should be performed.

In the present study, we demonstrated that PBM plays a key role in normal hippocampal neurogenesis, and that normal neurogenesis induced by PBM prevents SE-induced hippocampal neuronal loss. Thus, PBM is a novel approach to prevent seizure-induced neuronal degeneration, for which light devices may be developed in the future.

## Materials and methods

### Animals

The study experiments were approved by the Dankook University Medical School Research Institutional Animal Care and Use Committee (DKU-19-001) and performed in compliance with the National Institutes of Health guidelines for animal research. And we confirm that all experimental procedures are reported in accordance with ARRIVE guidelines for the reporting of animal experiments. The animals were housed under a 12-h light/dark cycle with ad libitum access to food pellets and water.

### SE animal model

We created a C57/BL6 male mouse SE model, as previously described^30^. Seven-week-old mice received 2 mg/kg of scopolamine methyl bromide (intraperitoneally [i.p.]; S8502; Sigma-Aldrich, St. Louis, MO, USA) and terbutaline hemisulfate salt (2 mg/kg, i.p.; T2528; Sigma-Aldrich) to block the peripheral effects of pilocarpine and dilate the respiratory tract to reduce mortality, respectively. After 30 min, the muscarinic agonist pilocarpine hydrochloride (320 mg/kg, freshly prepared, i.p.; P6503; Sigma-Aldrich) was injected to induce SE. Seizure activity was scored using Racine’s classification^31^. Only mice with a Racine’s scale stage of 5 or above for behavioral seizures were selected for the experiments.

### PBM treatment

PBM irradiation was performed using an 830-nm diode laser (Fig. 1A; Wontech, Daejeon, South Korea) that penetrated the scalp and skull to reach the brain in the SE mouse model. PBM was anatomically irradiated to the scalp just above the hippocampus. As a result of measuring the laser transmittance for the scalp and skull, 63.7% of the original energy of the laser light reaches the neocortex area under the scalp^32^. During laser treatment, mice were immobilized using a restrainer and irradiated with PBM after 4 h of SE (Fig. 1B). Mice did not show any symptoms of depression or anxiety due to restraint (Fig. S1). The two PBM treatment groups were irradiated on a single and 5 consecutive days, respectively. The distance between the scalp and fiber end was 5 cm, the power density was 50 mW/cm^2^ (maintained for 12 min), and the total radiant energy at the brain (spread over the entire head) was 22.9 J/cm^2^ (Fig. 1C). The light source power density was measured using a laser power meter (PD-300 and VEGA power meter; Ophir, Darmstadt, Germany). The laser specifications are described in detail in Table 1.

**Table 1:** Specifications for laser parameters.

**Optical parameters**	
Central wavelength (nm)	830
Wavelength tolerance (nm)	± 10
Max output power (W)	1
Spectral width (FWHM, nm)	< 3.0
Wavelength temp. coefficient	0.3
**Fiber parameters**	
Fiber core diameter (um)	60
Numerical aperture	0.14
Fiber connector	SMA 905
**Treatment parameters**	
Irradiance power at skin (mW/cm^2^)	50
Exposure duration (s)	720
Radiant exposure at skin (J/cm^2^)	36
Laser transmission ratio to skin and skull (%)	63.7
Irradiance power at brain (mW/cm^2^)	31.8
Distance to the head from the end of a fiber (cm)	5
Focused beam spot size (mm^2^)	14.01
Number of points irradiated	1 or 5
Radiant exposure at brain (J/cm^2^)	22.9

### Immunohistochemistry

To evaluate cell proliferation and neurogenesis in the hippocampus of trained mice, BrdU (50 mg/kg b.w.; Sigma-Aldrich) was administered daily by i.p. injection for 3 consecutive days before pilocarpine injection. At 7, 14, and 21 days, the mice were transcardially perfused with 4% buffered paraformaldehyde (PFA; Daejung, Korea). The mice brains were decapitated, fixed overnight, and cooled with 4% PFA in phosphate-buffered saline (Corning, Corning, NY, USA). After fixation, the brain samples were cryoprotected in 30% sucrose dissolved in phosphate-buffered saline (PBS), and frozen in OCT compound (Sakura, Tokyo, Japan) for storage at – 80 °C. Then, 18-μm-thick coronal sections of the brain were created using a cryostat microtome (Leica Microsystems, Nussloch, Germany). Hippocampal tissue sections were permeabilized with 0.5% Triton X-100 for 5 min. The tissues were blocked with 10% bovine serum albumin (BSA) and incubated overnight at 4 °C with the following primary antibodies: rabbit anti-DCX (ab18723, 1:1000; Abcam, Cambridge, UK), rabbit anti-Ki67 (ab15580, 1:500; Abcam), and mouse anti-NeuN (MAB377, 1:500; Sigma-Aldrich). For BrdU immunohistochemistry, sections were pre-treated for 15 min with cold 2 M HCl and 2 M HCl at 37 °C for 30 min to denature the DNA. After rinsing in PBS, they were incubated with the primary anti-BrdU antibodies. After treatment with primary antibodies, the tissues were incubated with secondary antibodies conjugated with Alexa Fluor 488-conjugated anti-rabbit IgG (A11008; ThermoFisher Scientific, Waltham, MA, USA) and Alexa Fluor 555-conjugated anti-mouse IgG (A21422; ThermoFisher Scientific) for 1.5 h at room temperature. Then, the sections were incubated with secondary antibodies and stained with DAPI (D9542, 1:1,000; Sigma) for 10 min to visualize the nuclei. The immunostained tissues were mounted with Vectashield antifade mounting medium (H-1000; Vector Laboratories Inc., Burlingame, CA, USA). Alexa Fluor 488- (excitation, 488 nm; emission, 520 nm) and Alexa Fluor 555 (excitation, 561 nm; emission, 568 nm)-labeled tissues were imaged using a confocal microscope (FV3000; Olympus, Tokyo, Japan). Brain tissue was quantified on the basis of six 18-µm-thick coronal sections. A Z-stack depth of 15 μm was used, with 1-μm intervals. At least five individual brain samples per group were analyzed and three slices per brain samples were stained. Data are presented as mean number of cells/section ± standard error of mean (SEM). The researcher conducting the confocal imaging and the assessor performing the analysis of immunohistochemistry were blinded to the group assignment.

### Statistical analysis

The data are presented as mean ± SEM. Statistical analysis was performed using one-way analysis of variance (ANOVA) followed by the Bonferroni test. The analyses were performed using GraphPad Prism version 7.04 (GraphPad Software, La Jolla, CA, USA). P-values < 0.05 were considered significant.

## Supplementary Information

Supplementary Figure S1.

## Data Availability

The datasets generated and/or analyzed during the current study are available from the corresponding author on reasonable request.
