# Supplementary material for: Photobiomodulation regulates adult neurogenesis in the hippocampus in a status epilepticus animal model
Source: Sci Rep. 2022 Sep 9;12:15246. doi: 10.1038/s41598-022-19607-5 (PMC9463127; doi:10.1038/s41598-022-19607-5)
Supplement: Supplementary file 1 — Supplementary Figure S1. [file 41598_2022_19607_MOESM1_ESM.docx]

**Supplementary materials**

**
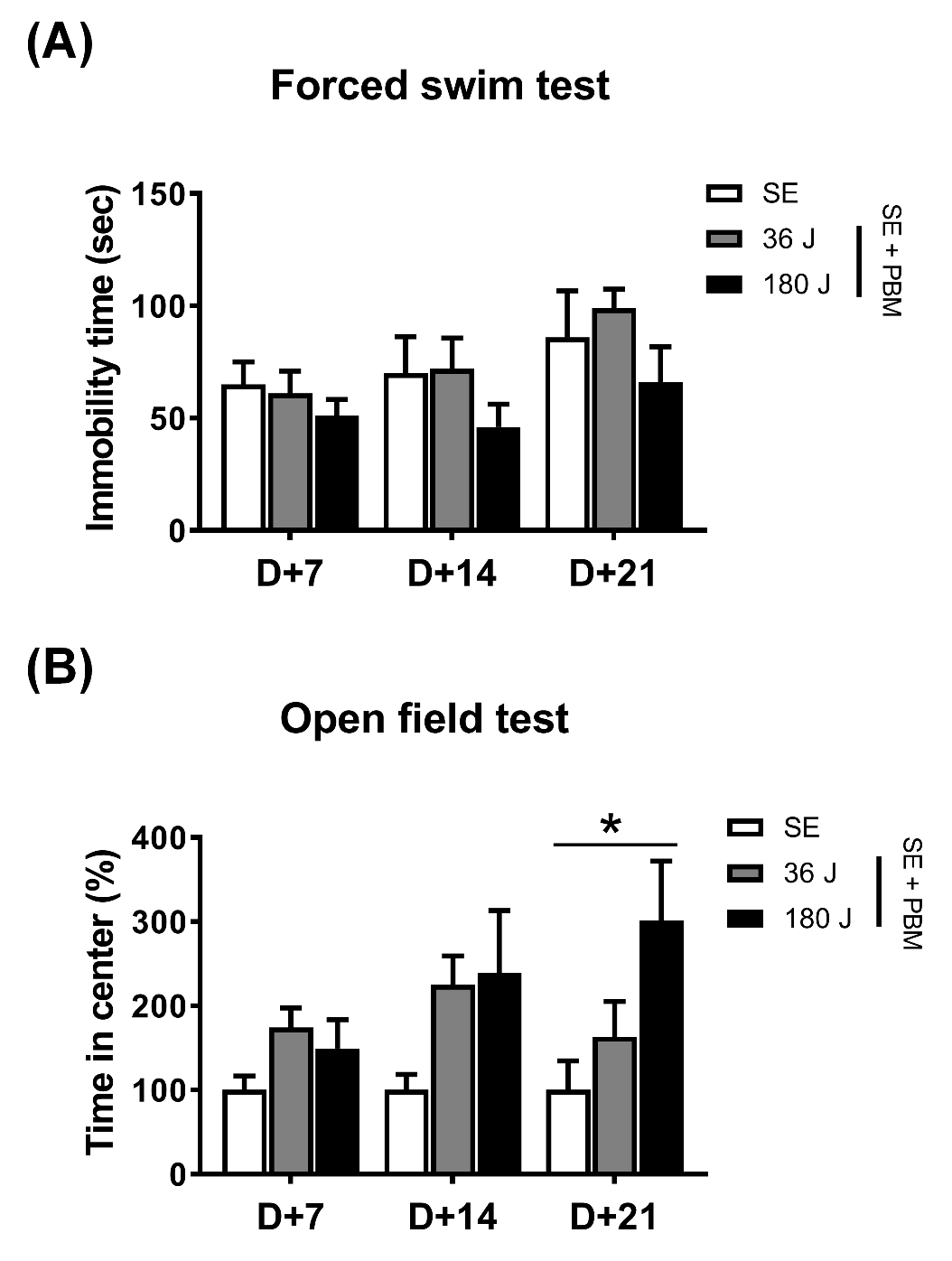
**

Figure S1. Forced swim test and open field test results. (A) Forced swim test; immobility time (B) Open field test; Time in center (*p < 0.05 relative to D+21 SE).vvvvvvvvvv
